# Imunological and clinical characteristics in children with polyarteritis nodosa: a retrospective study over the last 20 years

**DOI:** 10.1186/1546-0096-9-S1-P91

**Published:** 2011-09-14

**Authors:** Masa Vikic Topic, Ivan Malcic, Danica Batinic, Danko Milosevic, Mandica Vidovic, Katarina Starcevic, Kristina Potocki, Branko Malenica, Marija Jelusic-Drazic

**Affiliations:** 1 University Hospital Centre Zagreb, University of Zagreb School of Medicine, ZAGREB, Croatia

## Aim

Analysis of polyarteritis nodosa (PAN) characteristics such as laboratory parameters, affected organs, treatment modalities and disease outcome.

## Methods

Our study includes all children aged 1-18 with PAN diagnosed according to EULAR/PRES/PRINTO criteria at Department of Paediatrics, University Hospital Centre Zagreb, Croatia, during the period of 1991-2010.

## Results

PAN was diagnosed in 12 patients (6 girls and 6 boys). The share of PAN amongst all vasculitides was 4%. The mean age at disease onset was (±SD) 11.33±3,08 years. Systemic PAN was diagnosed in 7 children (58%), microscopic polyangiitis in 3 (25%), cutaneus PAN in 2 (17%) and classic PAN in 0 (0%). The most consistent symptoms were skin involvement (90%) and arthritis/arthralgia (60%). The CNS was affected in 40% of patients. ESR and CRP were elevated in all patients. Antineutrophil cytoplasmic antibodies were elevated in 3 patients (25%). Antistreptolysin O was elevated in 4 patients (25%). The relation between the severity of skin involvement and involvement of other organs was not found. Therapy mode for all patients was corticosteroids. Immunosuppressive drugs and Rituximab (anti-CD20) were used as additional therapy for patients with severe symptoms. Two patients with microscopic polyangiitis died due to chronic renal and pulmonary failure during the follow-up.

## Conclusion

In comparison to available studies, we found a difference in distribution of childhood polyarteritis nodosa as well as some clinical characteristics (e.g. higher prevalence of neurological symptoms), while other researched features, laboratory and treatment, were similar.

